# Medicine

**Published:** 1911-06

**Authors:** J. M. Fortescue-Brickdale


### Progress of the Medical Sciences.

### MEDICINE.

**The Measurement of Arterial Pressures.**—All measurements of arterial pressures now in use depend upon the compression of an artery by an armlet or cuff into which air can be pumped, and a manometer or aneroid dial to record the pressure at any given point during the observation. But not only are there several points in the arterial cycle in which this pressure may be noted, but also there are several methods of ascertaining the exact moment in time at which these points occur. Thus, as is indicated by Gibson,[3] there is the maximum or systolic

[2] *Rep. Royal Soc. Med., Med. Sect.*, 1909.  [3] *Lancet*, 1910, ii. 1699.

pressure or point at which the pulsation of the artery is arrested, the minimum or diastolic pressure or point at which the column of mercury or the needle of the aneroid shows the widest oscillations, and the mean pressure or point midway between these two. The difference between maximum and minimum pressures is the " pulse pressure," and the ratio between the pulse pressure and the maximum or systolic pressure is the " co-efficient of pressure."

There are four ways in which the occurrence of the maximum, minimum, and mean pressures can be determined in practice.[1] (1) The palpatory or tactile, in which the obliteration of the arterial pulse is determined by the finger. (2) The graphic, in which the obliteration of the pulse is determined by an ordinary sphygmograph fastened over the artery instead of the observer's finger. (3) The oscillatory, in which variations in the oscillation of an indicator are noted by the eye. (4) The auscultatory, in which the sounds produced in the artery when the cuff or armlet is inflated or deflated are observed by means of some form of stethoscope.

These four methods may now be described in more detail.

(1) The palpatory or tactile method, in spite of its simplicity, is open to serious objection. The personal factor, as pointed out by Oliver,[2] cannot be sufficiently eliminated. Even trained observers may make an error of 10 mm. of mercury in estimating the point at which pulsation in the artery ceases, while in less skilled persons the limits of error are necessarily much wider.

(2) The graphic method, which has been employed by Janeway and later by Masing and Sahli, requires two observers, one to look after the manometer and the other to watch the sphygmograph tracing; moreover, there are oscillations due to the movement of the sphygmograph needle, and the points of correspondence between manometer pressures and the pulse tracings are not easy to determine.[3]

(3) According to Oliver,[4] the significance of the variations in oscillation of the manometer or aneroid needle is not absolutely certain. The point of reappearance of the oscillations when the armlet is deflated is certainly a measure of the maximum or systolic pressure, but the point at which it again disappears as the deflation is continued is not so definitely significant. In his opinion, and also in the opinion of Ettinger and Gittings, the lower limit of oscillation represents the true diastolic pressure, a point which others believe is represented by the

[1] Goodman and Howell, *Univ. Penna. Med. Bull.*, 1910, xxiii. 469.

[2] *Proc. Roy. Soc. Med.* (*Med. Section*), 1910, iii. 207.

[3] Hollmann, *St. Petersb. Med. Wchnschr.*, 1910, xxxv. 560.

[4] *Quart. J. Exper. Physiol.*, 1911, iv. 47.

point at which the oscillations are widest in range (optimum oscillation). Oliver believes that it will be shown hereafter that the point of optimum oscillation or maximal excursion of the indicator represents the *mean* arterial pressure.

(4) The auscultatory method was first described by Korotkow in 1905, and although the exact description and significance of the sounds heard have been variously given by several observers, there is in the main a considerable body of opinion in favour of its being the most accurate and useful of all the methods as yet described. Goodman and Howell [1] thus describe the procedure. An ordinary armlet connected with a manometer is placed over the brachial artery, and the sounds are auscultated with a stethoscope over the same artery, just below the point of compression, care being taken not to press on the artery. The pulsation having been arrested by pumping up the armlet, the air in it is gradually released, and a series of sounds are heard which have been divided into a varying number of "phases" by different observers. Krylow recognised three; the first sound a "loud, clear-cut, snapping tone," then a murmur, and lastly a second sound, duller than the first, which becomes "muted," and finally disappears. The last of these phases was by Ettinger and Korotkow further subdivided into two portions, the first of which resembled the original first sound, but was less well marked, and the second (named the fourth phase) consisted of a duller note. Finally a fifth phase, in which all sounds disappeared, was added to the preceding four.

The first four phases are described by Oliver [2] as a series of "throbs," characterised (1) by sharpness, (2) by murmurishness, (3) by loudness, (4) by dulness. He considers that the third is the least constant, being frequently absent or too short for detection.

The causes of the various phases are probably as follows. It is practically certain that the first phase is due to the inrush of blood into the empty artery below the point of constriction. It represents, therefore, the maximum or systolic pressure. The second phase is possibly produced by eddies in the blood current as the artery gradually fills up. The third and fourth phases are practically transitional; but, as will be seen, considerable importance is attached to the third phase in clinical observations, because its character will give a clue to the degree to which the artery below the armlet has become collapsed, and the rapidity with which it refills. The fifth phase, or as Oliver puts it the lower limit of the range of the throb, is thought to indicate the diastolic pressure, but there is some uncertainty still on this point.

In normal persons, with a pulse pressure of 45 mm. of

[1] *Loc. cit.* [2] *Loc. cit.*

mercury, Goodman and Howell determined the average pressures as follows :—

| | |
|---|---|
| First phase .. .. .. .. .. .. | 14 mm. |
| Second phase .. .. .. .. .. .. | 20 mm. |
| Third phase .. .. .. .. .. .. | 5 mm. |
| Fourth phase .. .. .. .. .. .. | 6 mm. |

Oliver, instead of using an ordinary stethoscope, has devised a small tambour, which is *lightly* buckled on to the bend of the elbow, with tubes leading to each of the observer's ears. He also confirms the auditory method by a visual one, in which the same or a second similar tambour is buckled *tightly* on to the thick part of the forearm, and connected with a small indicating tube filled with coloured spirit. The oscillations of the column of spirit (maximum, minimum, and optimum) are noted.

**Clinical Interpretation of Blood Pressures.**—Fischer[1] has shown that in anæmia from various causes the second phase (murmur) is long and well marked, but the third phase is diminished. There is prolongation of the fourth phase, and the lower limit (fifth phase) shows a low mercurial reading. A number of observers agree that in cases of cardiac insufficiency the second and third phases are weakened or absent. Some believe (Ettinger) that the absence of the third phase only shows a moderate degree of insufficiency, and that in the extreme grades the second, third and fourth all disappear. Fischer noted that in arterio-sclerosis the third phase was very distinct; and this was practically confirmed by Goodman and Howell, who also found that when the second and third phases were not prolonged, in spite of arterial thickening, the circulation shows signs of failing. They also made the interesting observation that in aortic incompetence the fifth phase ceased to exist, that is the murmur or " third " continued after the pressure on the artery had been released below the point of diastolic pressure.

When a weakened heart is improving under treatment the sequence of phases tends to return to normal, so that the effect of any therapeutic measures may be gauged.

**Normal and Abnormal Pressures.**—Lowsley[2] has made observations on the effect of exercise on normal blood pressures. He finds that all forms of exercise cause a rise in both systolic and diastolic pressures, but that the rise is greater in the former. The pulse pressure is thus increased, which may indicate stronger cardiac action as well as increased pulse rate. After a certain period reaction sets in and the pressures fall below normal; the more exhausting the exercise the longer this fall will persist. The systolic pressure shows the greatest fall, and

[1] *Deutsche Med. Wchnschr.*, 1908, p. 1141.

[2] *Am. J. Physiol.*, March, 1911.

thus the pulse pressure falls also. Moderate exercise of a long period fails to influence pressures so much as a short and violent effort.

Gibson[1] quotes as extreme abnormal pressures 300 mm. systolic and 260 mm. diastolic in interstitial nephritis with arterial degeneration, 150 mm. systolic and 70 diastolic in aortic regurgitation, 270 mm. systolic and 80 mm. diastolic in heart block.

In acute infections (especially typhoid) there is an early rise of blood pressure and an early fall, and in lead poisoning and gout the pressure is also high. Arterio-sclerosis does not necessarily involve high arterial pressures. Different observers have given 35 to 50 per cent. as the number in which they remained low. In anæmia, asthma, and myxœdema the pressure is high; in cases of amyloid kidney and Graves's disease it varies, but is usually low; in phthisis and Addison's disease it is low.

J. M. Fortescue-Brickdale.

